# From Basal Sources to Top Predators: Isotopic Evidence of Trophic Organization in the Francisco Coloane Coastal Marine Protected Area

**DOI:** 10.3390/biology15191699

**Published:** 2026-09-23

**Authors:** Lisette Zenteno-Devaud, Gabriela V. Aguirre-Martínez, Matías Cárcamo-Chávez, Paola Acuña-Gómez, Jorge Acevedo, Claudio A. Moraga, Lautaro Oyarzún-Galaz, Pedro Valenzuela, Anelio Aguayo-Lobo

**Affiliations:** 1 División de Investigación y Aplicaciones Nucleares, Comisión Chilena de Energía Nuclear (CCHEN), Nueva Bilbao 12501, Santiago 7600713, Chile; 2 Centro de Estudios del Cuaternario Fuego-Patagonia y Antártica (CEQUA), Avenida El Bosque 0296, Punta Arenas 6200000, Chile; matias.carcamo@umag.cl (M.C.-C.); paola.acuna@cequa.cl (P.A.-G.); jorge.acevedo@cequa.cl (J.A.); claudio.moraga@cequa.cl (C.A.M.); lautaro.oyarzun@cequa.cl (L.O.-G.); pedro.valenzuela@cequa.cl (P.V.); aaguayo@inach.cl (A.A.-L.); 3 Facultad de Ciencias de la Salud, Universidad Arturo Prat, Avenida Arturo Prat 2120, Iquique 1110939, Chile; 4 Magíster en Bioinformática, Facultad de Ingeniería, Universidad de Magallanes, Av. Pdte. Manuel Bulnes 01855, Punta Arenas 6200985, Chile; 5 Departamento Científico, Instituto Antártico Chileno (INACH), Plaza Muñoz Gamero 1055, Punta Arenas 6210427, Chile

**Keywords:** ecosystem connectivity, stable isotopes, Coastal Marine Protected Area, trophic convergence

## Abstract

Coastal food webs are sustained by two contrasting energy channels—a fast pelagic pathway fueled by phytoplankton and a slow benthic pathway built on detrital processing—yet whether these channels remain segregated or become progressively coupled as energy moves toward higher consumers is still poorly resolved, particularly in remote, environmentally variable systems. We tested these competing scenarios—channel compartmentalization versus channel coupling—using carbon (δ^13^C) and nitrogen (δ^15^N) stable isotopes across a four-level food web in the Francisco Coloane Marine Coastal Protected Area, a subantarctic system in the Strait of Magellan, Chile. Lower-trophic consumers showed strong isotopic differentiation, tracking access to distinct basal resources, but this segregation gave way to substantial niche convergence at intermediate trophic levels, where overlap probabilities exceeded 60%. Macroalgal-derived carbon dominated assimilation across resident consumers (50–80%), while sediment organic matter acted as an integrative pool rather than an independent pathway, blending detrital inputs from multiple sources. The clearest evidence for channel coupling came from the squat lobster *Grimothea gregaria*: its pelagic and benthic life stages occupied opposite ends of the isotopic gradient, showing that a single species can physically cross channels that appear isotopically segregated at the community level. Together, these results indicate that benthic and pelagic energy pathways in this protected area are not independent but progressively integrated as energy moves up the food web—a pattern with direct implications for how spatial management should treat habitat connectivity in coastal marine reserves.

## Simple Summary

Coastal food webs are typically organized around two basal energy channels—a fast pelagic pathway and a slower benthic one—but the extent to which these channels remain segregated as energy is transferred to higher trophic levels is rarely tested in remote systems. Using carbon and nitrogen stable isotopes across a four-level food web in the Francisco Coloane Marine Coastal Protected Area (Strait of Magellan, Chile), we found that basal resource heterogeneity structured isotopic niches strongly at lower trophic positions but gave way to substantial niche overlap and redundancy at intermediate levels. Macroalgae dominated assimilated carbon across resident consumers, while sediment organic matter functioned as an integrative rather than an independent basal source. These results support a partially coupled trophic architecture, in which channel differentiation at the food-web base is progressively integrated rather than propagated toward higher consumers, with direct implications for how energy connectivity should inform the management of this protected area.

## 1. Introduction

Understanding how energy flows through marine food webs is fundamental for evaluating ecosystem organization and responses to environmental change [1,2]. Food webs are commonly represented as simplified trophic chains linking primary producers to higher consumers; however, such simplifications may overlook important mechanisms governing resource partitioning and trophic organization [3,4]. In coastal ecosystems, primary production is frequently transferred through contrasting basal energy channels that differ in their spatial origin, turnover rates, and trophic dynamics [1,5]. Fast pelagic pathways are typically associated with phytoplankton-derived production characterized by rapid turnover and short transfer pathways, whereas slow benthic pathways rely on detrital processing and benthic primary producers, resulting in longer energy residence times within food webs [1,6].

Importantly, these pathways do not necessarily represent direct and independent links between individual primary producers and consumers. In particular, the benthic compartment can integrate organic matter originating from multiple basal sources through detrital processing. Senescent or fragmented macroalgal and phytoplankton material may be exported, buried, and microbially reworked before being assimilated by consumers [7,8]. This processing alters the composition of organic matter, and surface sediments consequently represent an integrative pool containing contributions from multiple primary producers rather than a single basal source.

The extent to which consumers couple to these pathways can strongly influence trophic overlap, niche differentiation, and the broader organization of marine communities [9,10]. When consumers rely on similar basal resources, trophic overlap and redundancy tend to increase [11,12]. Conversely, differential coupling to benthic and pelagic pathways may favor trophic segregation and niche differentiation among taxa with contrasting habitat use and feeding strategies [2,5]. Spatial segregation of basal resources can further limit the extent to which consumers integrate energy across habitats, promoting the emergence of partially structured trophic pathways linked to distinct energy sources. Consequently, trophic redundancy is not fixed but emerges from the interaction between resource heterogeneity, habitat structure, and consumer foraging ecology, thereby influencing food-web organization [2].

Stable isotope analyses provide high-resolution quantitative information on the assimilation of basal sources and trophic connectivity within food webs [13,14,15,16]. Carbon isotopes (δ^13^C) are particularly useful for identifying basal carbon sources because they reflect spatial variation in primary production and habitat origin [17], whereas nitrogen isotopes (δ^15^N) provide reliable information on trophic position [18,19]. Beyond basic isotopic ratios, methodological advances including Bayesian mixing models and isotopic niche metrics now allow detailed evaluation of dietary breadth, trophic overlap, niche segregation, and redundancy patterns within food webs [20,21,22].

The ecological interpretation of isotopic patterns depends strongly on both the temporal scale of isotopic integration and the spatial ecology of consumers. In this context, resident species are particularly informative because they integrate isotopic signals derived from local food webs over time, thereby providing a spatially explicit representation of the dominant basal resource pathways sustaining regional trophic networks [13,23]. In contrast, incorporating both resident and non-resident consumers into isotopic niche analyses allows evaluation of trophic overlap, niche segregation, and trophic redundancy across the broader assemblage [20,21,22]. Consequently, species occupying different habitats and trophic roles may exhibit distinct positions within isotopic space, reflecting differences in resource use, habitat coupling, and the relative contribution of benthic and pelagic primary food sources.

These approaches are particularly valuable in remote and environmentally complex regions such as southern Chilean Patagonia, where direct observations of trophic interactions remain limited [24,25]. The Francisco Coloane Coastal Marine Protected Area (FC-MCPA), located in the westernmost sub-basin of the Strait of Magellan, is characterized by the interaction of Pacific and Atlantic waters, freshwater discharge, strong tidal mixing, and complex coastal geomorphology [26,27]. These processes generate spatially heterogeneous habitats and basal resource pools that may support contrasting benthic and pelagic energy pathways [2,5,23].

The ecological consequences of such pathways depend not simply on their coexistence, but on the extent to which they become coupled across the food web. Under the structural-asymmetry framework, spatially segregated primary production supports distinct “fast” (pelagic) and “slow” (benthic) energy channels, whose differential coupling across trophic levels can shape patterns of niche segregation and trophic redundancy [2,6].

In the Strait of Magellan, connectivity between benthic and pelagic compartments has primarily been inferred from mass-balance ecosystem models [25,28] and, more recently, from topological network analyses identifying consumers such as the Patagonian blenny *Eleginops maclovinus*, polychaetes, and the Fuegian sprat *Sprattus fuegensis* as potential links between benthic and pelagic production and higher trophic levels [29]. However, these approaches cannot quantify the relative contribution of alternative energy pathways to consumer biomass or determine whether their coupling changes systematically across trophic levels.

Isotope-based descriptions of regional food webs remain scarce, being restricted to the neighboring Beagle Channel [23] and a single winter assessment in a nearby Patagonian fjord, which suggested comparatively weak benthic–pelagic coupling under low-productivity conditions [30]. Consequently, it remains unknown whether, and how, coupling between isotopically distinct benthic and pelagic energy pathways changes across the trophic hierarchy of a subantarctic marine protected area. Here, we address this gap by providing an isotope-based assessment of energy–channel coupling across the FC-MCPA food web, from basal resources to top predators.

In this study, we used δ^13^C and δ^15^N stable isotope analyses to investigate how contrasting basal resource pathways structure trophic organization within the Strait of Magellan ecosystem. We focused on resident consumer species spanning pelagic and benthic habitats to evaluate how locally integrated energy sources are assimilated across taxa with different ecological strategies. In parallel, isotopic niche structure was assessed across the broader trophic assemblage, including non-resident consumers, to examine patterns of trophic overlap, niche segregation, and isotopic redundancy within the regional food web. Specifically, we aimed to: (1) characterize isotopic differentiation among resident consumers associated with alternative benthic and pelagic primary food sources; (2) quantify isotopic niche structure and trophic overlap across the broader consumer assemblage; and (3) evaluate whether the coupling between benthic and pelagic energy channels changes systematically with trophic position within the FC-CMPA food web.

We tested whether the FC-MCPA food web is structured by the progressive coupling of “fast” pelagic and “slow” benthic energy channels across trophic levels [2,6]. Under the channel-coupling hypothesis (H1), isotopic niche segregation should decrease, while resource overlap and redundancy increase toward higher trophic levels as mobile consumers integrate otherwise distinct energy pathways. Alternatively, under the compartmentalization hypothesis (H2), benthic and pelagic channels should remain segregated across the food web, with consumers consistently associated with a dominant basal pathway.

## 2. Materials and Methods

### 2.1. Study Area and Field Sampling

The Magellan Strait is part of the Patagonian Cold Estuarine System and forms a natural corridor connecting the South Pacific and South Atlantic Oceans at the southern tip of South America. The study area encompasses the Francisco Coloane Coastal Marine Protected Area (FC-CMPA), covering approximately 2917 km^2^ (Figure 1). Field sampling was carried out at five sites across the FC-CMPA—Choiseul Bay, southern Carlos III Island, Paso Shag, Monmouth Island, and Cayetano Island—spanning nearshore, intertidal/subtidal, and colony habitats (Figure 1). Between 12 January and 20 April 2023, a total of 15 consumer taxa and six primary food sources were obtained from these locations (Figure 1; Table 1).

Hair samples of Southern sea lion *Otaria byronia* were obtained from biopsy samples collected using a crossbow equipped with biopsy darts (175 lb draw weight; (Bruin Outdoors, Ambush 425; Dothan, AL, USA)), with hair subsequently separated from the skin tissue in the laboratory. Skin samples of humpback whale *Megaptera novaeangliae* were collected using the same biopsy dart system. Blood samples from *Spheniscus magellanicus* (Magellanic penguins) were collected from the brachial vein using sterile 3 mL syringes and 23-gauge needles (Cranberry^®^), with a total volume of 0.5 mL collected per individual. Fish, crustaceans, and cephalopods were collected using a trammel net (inner mesh size 3 in; outer panels ~10 in) deployed from small vessels (5–14 m) operating near the seafloor. To avoid potential isotopic variability across different body structures, muscle tissue sampling was obtained from: the abdominal region for fish, legs for crustaceans, and arms for cephalopods. Crustacean exoskeletons were meticulously removed before dissection to prevent contamination from non-muscle tissues [31]. Additionally, the squat lobster *Grimothea gregaria* specimens were categorized by their habitat-associated morphs rather than size. The benthic form represents seafloor-associated adults, whereas the pelagic form designates a post-larval or juvenile stage inhabiting the water column. These classifications are not strictly size-dependent, as pelagic individuals can attain body sizes comparable to those of benthic adults [32]. The polychaete *Platynereis australis* was obtained from the holdfasts of giant kelp (*Macrocystis pyrifera*), which were collected both manually and by SCUBA diving at depths < 20 m. Intertidal invertebrates, including the limpet *Nacella magellanica* and the mussel *Perumytilus purpuratus*, were hand-picked during diurnal low tides. Consumers were classified as resident or non-resident based on their site fidelity and home-range extent relative to the FC-CMPA. Resident taxa, *Eleginops maclovinus*, *Patagonotothen* sp., *Enteroctopus megalocyathus*, *Sprattus fuegensis*, *G. gregaria* (benthic and pelagic phases), *Lithodes santolla*, *N. magellanica*, *P. purpuratus*, and *P. australis*, complete their life cycle within, or show high site fidelity to, the study area and were therefore included in the resource-assimilation (mixing-model) analyses. Non-resident taxa, *O. byronia*, *Merluccius australis*, *M. novaeangliae*, *S. magellanicus*, and *Oncorhynchus* sp. are highly mobile species that forage well beyond the spatial extent of the FC-CMPA.

Benthic sources included intertidal and subtidal macroalgae, hand-harvested during low tide, and surface sediment organic matter (OM), sampled from the holdfast zones of *M. pyrifera*. Suspended particulate organic matter (POM) was collected from the sea surface layer (0.5 m depth) using a 5 L Go-Flo sampling bottle General Oceanics, Miami, FL, USA), then filtered (~2.0 L per sample) through pre-combusted (4 h at 550 °C) 47 mm GF/F glass fiber filters (0.7 μm pore size; Cytiva, Little Chalfont, UK)), following protocols for organic matter retention in marine environments [33]. Phytoplankton samples were obtained using horizontal tows with a WP-2 net (Hydro-Bios, Kiel-Altenholz, Germany) equipped with 100 μm mesh, targeting the microplankton fraction. All biological samples were gently rinsed with distilled water to remove debris and salts, placed in sterile plastic bags by taxon or food source, and immediately stored at –2 °C until laboratory processing for stable isotope analysis.

**Table 1:** Summary of δ^13^C and δ^15^N values, trophic position, and width isotopic niche areas (SEAc) of consumers and sources from the food web in the Francisco Coloane Coastal Marine Protected Area.

Species	Trophic Position (Mean ± SD)	*n*	δ^13^C ‰ (Mean ± SD)	δ^15^N ‰ (Mean ± SD)	SEAc ‰^2^	Feeding Strategies	References
**Non-resident taxa**							
*Otaria byronia*	3.9 ± 0.3	14	−16.0 ± 0.8	18.2 ± 1.0	2.9	Top predator	Alonso et al. [34]
*Merluccius australis*	4.3 ± 0.2	6	−17.0 ± 0.4	19.7 ± 0.7	0.4	Demersal predator	Dunn et al. [35]
*Megaptera novaeangliae*	3.0 ± 0.4	16	−18.0 ± 0.3	15.5 ± 1.3	2.7	Planktivore/Filter feeder	Acevedo et al. [36]
*Spheniscus magellanicus*	2.8 ± 0.0	10	−18.3 ± 1.5	17.0 ± 0.4	1.5	Piscivore/Planktivore	Pütz et al. [37]
*Oncorhynchus* sp.	3.0 ± 0.2	8	−18.7 ± 0.5	14.3 ± 0.5	0.6	Predator	Ciancio et al. [38]
**Resident taxa**							
*Enteroctopus megalocyathus*	3.5 ± 0.4	8	−16.8 ± 0.9	16.9 ± 1.2	3.1	Benthic predator	Ibáñez & Chong [39]
*Eleginops maclovinus*	3.7 ± 0.1	10	−14.7 ± 0.4	17.4 ± 0.5	1.2	Benthic generalist	Licandeo et al. [40]
*Patagonotothen* sp.	3.8 ± 0.1	9	−14.1 ± 0.9	17.9 ± 0.3	0.4	Benthopelagic carnivore	Laptikhovsky [41]
*Sprattus fuegensis*	2.3 ± 0.6	10	−16.5 ± 0.9	12.9 ± 1.1	1.2	Zooplanctivore	Montecinos et al. [42]
*Grimothea gregaria (benthic)*	2.4 ± 0.1	10	−15.6 ± 0.7	13.3 ± 0.4	0.9	Scavenger/Benthic predator	Romero et al. [43]
*Grimothea gregaria (pelagic)*	1.3 ± 0.2	14	−21.6 ± 1.1	9.5 ± 0.7	2.7	Planktivore/Pelagic filter feeder	Romero et al. [43]
*Lithodes santolla*	2.8 ± 0.1	9	−13.9 ± 1.0	14.4 ± 0.4	1.4	Scavenger/Generalist predator	Andrade et al. [44]
*Nacella magellanica*	2.0 ± 0.1	7	−14.2 ± 1.2	14.9 ± 0.9	1.1	Herbivore/Grazer	Rosenfeld et al. [45]
*Perumytilus purpuratus*	1.8 ± 0.1	7	−16.9 ± 0.7	13.5 ± 2.7	0.4	Suspension feeder/Filter feeder	Montory et al. [46]
*Platynereis australis*	1.8 ± 0.0	5	−13.9 ± 1.0	11.3 ± 0.2	0.7	Detritivore/Grazer	Cañete et al. [47]
**Primary food sources**							
POM	0.5	5	−29.4 ± 3.2	7.2 ± 0.6	–		
Surface sediment OM	0.3	5	−21.7 ± 0.7	10.3 ± 0.9	–		
Phytoplankton	0.0	5	−26.2 ± 0.1	3.3 ± 0.6	–		
*Ulva lactuca*	0.7	5	−11.9 ± 0.4	11.1 ± 0.4	–		
*Adenocystis utricularis*	0.1	5	−16.8 ± 0.3	10.5 ± 0.5	–		
*Macrocystis pyrifera*	0.3	5	−12.7 ± 0.5	10.3 ± 0.9	–		

### 2.2. Stable Isotope Sample Preparation and Analysis

In the laboratory, all samples were oven-dried at 60 °C for 36–48 h. Once dried, tissues were ground into a fine powder using a mortar and pestle (see Table 1). Samples containing calcium carbonate—such as POM filters, surface sediment OM, macroalgae, and polychaetes—were acidified with 1N HCl for 4 h until CO_2_ release ceased, following the protocol of Newsome et al. [31]. These acidified samples were then dried at 60 °C for 24 h. As acid treatment can alter δ^15^N values [48], a separate, non-acidified aliquot was used exclusively for δ^15^N analysis. Lipids were removed using a chloroform:methanol solution (2:1, *v*/*v*) according to the method described by Bligh and Dyer [49]. Carbon and nitrogen stable isotope ratios were determined using an elemental analyzer coupled to an isotope ratio mass spectrometer (Flash EA200 IRMS Delta Series, Thermo Scientific, Bremen, Germany) at the Laboratory of Biogeochemistry and Applied Stable Isotopes (Pontificia Universidad Católica de Chile, Santiago, Chile). Isotope values were expressed in δ notation according to the standard equation:

δX = [(R_sample/R_standard) − 1]

where X is either ^13^C or ^15^N, and R represents the ratio of heavy to light isotopes. Measurements were reported relative to the VPDB scale for δ^13^C and the atmospheric N_2_ (Air) scale for δ^15^N. Laboratory reference materials were used for instrument calibration, normalization, linearity assessment, and analytical quality control. Instrumental calibration and quality control were assessed using laboratory reference materials analyzed during the analytical sequence. Acetanilide was used to evaluate instrument linearity over a range of sample masses (0.273–1.528 mg; *n* = 17), yielding coefficients of determination of R^2^ = 0.9967 for nitrogen and R^2^ = 0.9931 for carbon. Additional laboratory reference materials, including atropine, caffeine, glutamic acid, PACC2, urea, aspartic acid, and sulfanilamide, were analyzed in triplicate to assess analytical reproducibility. The standard deviations of replicate measurements ranged from 0.048 to 0.158‰ for δ^15N and from 0.022 to 0.115‰ for δ^13^C. These replicate measurements were used to evaluate the consistency of isotope measurements across reference materials. Overall analytical precision, based on the standard deviation of internal laboratory standards, was ±0.18‰ for δ^15^N and ±0.30‰ for δ^13^C.

### 2.3. Trophic Position

Trophic position (TP) was estimated using the δ^15^N baseline approach proposed by Post [13]. Because the isotopic composition of primary producers can vary spatially and temporally, two locally occurring primary consumers, *Perumytilus purpuratus* and *Nacella magellanica*, were initially evaluated as potential isotopic baselines. Both species were assigned a baseline trophic position (TP_base_) of 2. *P. purpuratus* had a mean δ^15^N value of 11.16 ± 0.47‰ (*n* = 7), whereas *N. magellanica* had a mean δ^15^N value of 11.82 ± 0.17‰ (*n* = 7). Trophic position was calculated as:

TP_consumer = [(δ^15^N_consumer − δ^15^N_base)/Δ15N] + TP_base

where δ^15^N_consumer_ is the mean δ^15^N value of the consumer, δ^15^N_base_ is the mean δ^15^N value of the selected baseline organism, TP_base_ is the trophic position assigned to the baseline (2), and Δ^15^N is the trophic enrichment factor between trophic levels. Following Post [13], a Δ^15^N value of 3.4‰ was used.

Trophic-position estimates obtained using *P. purpuratus* and *N. magellanica* as alternative baselines were highly comparable, differing by approximately 0.19 trophic-position units. Given the lower variability in δ^15^N among the sampled *N. magellanica* individuals, and its suitability as a primary consumer integrating the isotopic signal of basal resources, *N. magellanica* was selected as the final baseline for all trophic-position calculations.

For community-wide analyses requiring discrete groupings, each consumer was assigned to the trophic level (TL) nearest its estimated TP (i.e., TP rounded to the nearest integer); primary food sources were treated as the food-web base and excluded from these comparisons.

### 2.4. Statistical Analysis

Stable isotope composition among primary producers was compared using a Permutational Multivariate Analysis of Variance (PERMANOVA; Anderson, 2001, 2017) [50,51] based on Bray–Curtis dissimilarities, with statistical significance assessed using 999 permutations under a reduced model. To minimize the influence of extreme values, δ^13^C data were converted to absolute values, and all isotopic data were square root transformed prior to analysis. This analysis was performed using PRIMER v7 with the PERMANOVA+ add-on [52]. A significance threshold of *p* ≤ 0.05 was used for all tests. To resolve heterogeneity among the six basal sources considered individually, normality of each source–isotope combination was first assessed using the Shapiro–Wilk test; because one combination departed from a normal distribution, sources were compared for each isotope using the non-parametric Kruskal–Wallis test, followed by Dunn’s post hoc test with Benjamini–Hochberg correction for pairwise comparisons (α = 0.05). Macroalgal producers were additionally compared against pooled non-macroalgal sources (POM, phytoplankton, and sediment OM) using paired *t*-tests for each isotope. Trophic structure, with an emphasis on trophic diversity and redundancy, was quantified using six community-wide Layman metrics calculated from the mean δ^13^C and δ^15^N values of consumer species [20]. Trophic diversity was characterized by: (1) Nitrogen Range (NR), which reflects the vertical extent of the trophic structure; (2) Carbon Range (CR), indicating the diversity of basal carbon sources, with wider ranges suggesting broader niche use; and (3) Mean Distance to Centroid (CD), representing overall trophic dispersion within the community. Trophic redundancy was assessed using: (4) Mean Nearest-Neighbor Distance (MNND), which describes the degree of trophic packing among species in isotopic space, with lower values indicating greater similarity in trophic roles; and (5) the Standard Deviation of Nearest-Neighbor Distance (SDNND), which reflects the evenness of trophic spacing, where lower values indicate more uniform niche distribution.

To estimate the isotopic niche width of each species, we calculated the standard ellipse area corrected for small sample sizes (SEAc), which provides a robust measure of core niche space [53]. Additionally, pairwise probabilistic niche overlap was quantified across species and trophic levels using the nicheROVER (version 1.1.2) package in R (version 4.6.1, R Core Team, Vienna, Austria) [54]. Overlap estimates were based on 10,000 Monte Carlo simulations of niche regions, defined at a 95% credibility level (α = 0.95), using species-specific δ^13^C and δ^15^N values as predictor variables.

Isotopic relationships between resident consumers and primary food sources were visualized using a δ^13^C–δ^15^N biplot, with mean values (±SD) plotted as group centroids. Consumer values were corrected using trophic discrimination factors of 3.4‰ for δ^15^N and 0.5 ± 0.2‰ for δ^13^C [13], and a convex hull polygon fitted around the corrected source values (chull function, R) defined the feasible isotopic mixing space. Proportional contributions of primary food sources to consumer diets were then estimated using Bayesian mixing models implemented in simmr (version 0.4.0) [55], applying the same discrimination factors; unlike the graphical approach, this framework incorporates uncertainty in source values and discrimination factors to yield probabilistic diet estimates [56]. Models were run for 10,000 iterations (1000 burn-in, thinning interval of 10) across four independent chains using simmr default priors. Model convergence was assessed for each consumer group using the Gelman–Rubin potential scale reduction factor (R-hat), with values close to 1 indicating that the Markov chains had converged.

## 3. Results

### 3.1. Trophic Organization Inferred from δ^13^C and δ^15^N Variability

In the food web of the Francisco Coloane Coastal Marine Protected Area, δ^13^C values ranged from −27.0‰ to −11.9‰, while δ^15^N values spanned from 3.3‰ to 19.9‰ (Table 1). Consumer isotopic composition varied widely among taxa, tracking differences in trophic position and habitat use. Among vertebrates, the fish Patagonian blenny *E. maclovinus* and *Patagonotothen* sp. were consistently enriched in both δ^13^C and δ^15^N, suggesting inshore foraging at high trophic levels. In contrast, the Fuegian sprat showed low δ^15^N enrichment, indicating a lower trophic position like the benthic stage of the crustacean *G. gregaria*. Compared to Southern sea lions, the humpback whale and the Magellanic penguin were relatively depleted in δ^13^C and δ^15^N, indicating more offshore foraging habits and a lower trophic position for both. Among omnivorous invertebrates, most species exhibited low enrichment in both isotopes, except for the red octopus *E. megalocyathus*, which showed elevated δ^13^C and δ^15^N values (Figure 2).

Estimated food web length was 4.04 ± 0.6 (Figure 2, Table 1), indicating a four-level trophic structure. Top trophic positions were occupied by the southern hake *Merluccius australis* and *O. byronia* (mean TP ± SD = 4.0 ± 0.3), followed by *S. magellanicus*, *E. megalocyathus*, *Patagonotothen* sp., *E. maclovinus*, and *M. novaeangliae*, and *Oncorhynchus* sp. (mean TP ± SD = 3.3 ± 0.5). Lower trophic levels included *P. australis*, *P. purpuratus*, *N. magellanica*, *L. santolla*, *S. fueguensis*, and both stages (benthic and pelagic) of *G. gregaria* (mean TP ± SD = 2.0 ± 0.5). Basal food sources exhibited pronounced, structured isotopic heterogeneity (PERMANOVA: F = 15.34, *p* = 0.001), delineating two isotopically distinct production channels. δ^13^C traced a clear benthic-to-pelagic gradient, from strongly enriched and highly variable macroalgal signatures (−16.8‰ in *A. utricularis* to −11.9‰ in *U. lactuca*) to markedly depleted values in POM, phytoplankton, and sediment organic matter (paired t-test, *p* < 0.001). δ^15^N followed a largely orthogonal axis, spanning 2.7‰ in phytoplankton to 11.3‰ in sediment organic matter, with pelagic sources significantly depleted relative to macroalgae and sediment (paired t-test, *p* < 0.001). This multivariate separation held at the level of individual sources: univariate tests (Kruskal–Wallis, applied given non-normal sediment δ^15^N; Shapiro–Wilk, *p* = 0.014) confirmed significant heterogeneity in both isotopes (δ^13^C: H = 28.94, df = 5, *p* < 0.001; δ^15^N: H = 25.92, df = 5, *p* < 0.001), with *U. lactuca* and POM forming the most divergent δ^13^C endmembers and *U. lactuca* and phytoplankton the most divergent in δ^15^N (Dunn’s test, Benjamini–Hochberg-corrected padj < 0.001). The single pair lacking resolution in either isotope was *A. utricularis* and sediment OM (padj > 0.05), a constraint carried forward into the source pool used in the mixing models (Table S1).

### 3.2. Isotopic Niche and Layman’s Metrics

When all consumer species were pooled (*n* = 15), the mean corrected standard ellipse area (SEAc) was 1.4 ± 1.0‰^2^, representing the average isotopic niche width (Figure 3, Table 1). SEAc values ranged from 0.4‰^2^ in *M. australis*, *P. purpuratus*, and *Patagonotothen* sp. to a maximum of 3.1‰^2^ in *E. megalocyathus*. No statistically significant differences in mean SEAc were observed across trophic levels (TL 4: 1.7 ± 1.8‰^2^; TL 3: 1.5 ± 1.1‰^2^; TL 2: 1.3 ± 0.9‰^2^). The probability of isotopic niche overlap between species is presented in Table 2. Substantial niche overlap (>60%) was observed within trophic levels 2 and 3. At trophic level 3, *M. novaeangliae* exhibited the highest probability of occupying the isotopic niche region of other species, particularly *Oncorhynchus* sp. (98.4%) and *E. megalocyathus* (63.7%). Within trophic level 2, the strongest overlap was observed between *S. fueguensis* and benthic *G. gregaria*, with a probability of 86.4%.

Analysis of Layman’s community-wide metrics revealed notable differences in trophic structure among levels. Trophic level 2 displayed the greatest ecological diversity, as evidenced by the widest ranges in δ^13^C and δ^15^N values, along with the highest mean distance to centroid (CD). These results indicate that species at this level exploited the broadest spectrum of carbon sources, occupied the most extensive vertical trophic space, and contributed significantly to overall trophic heterogeneity within the community (Table 3). In contrast, trophic level 3 exhibited the lowest mean nearest-neighbor distance (MNND), reflecting a higher degree of trophic redundancy. This suggests that species within this level shared more similar isotopic niches, indicating convergence in assimilated resources and reduced niche differentiation.

### 3.3. Contributions of Primary Food Sources to Resident Consumer Diets

MCMC convergence was satisfactory for all simmr mixing models, with Gelman–Rubin potential scale reduction factors (R-hat) ranging from 1.0006 to 1.0018 (maximum R-hat = 1.0018). The isotopic signatures of resident consumers were distributed across the isotopic space defined by the six primary food sources, with most taxa occupying the central region of the mixing polygon or lying close to its upper boundary (Figure 3). Most consumers overlapped primarily with the isotopic signatures of the macroalgal sources (*A. utricularis*, *M. pyrifera*, and *U. lactuca*), whereas the pelagic stage of *G. gregaria* exhibited the lowest δ^13^C and δ^15^N values among consumers, plotting closer to sediment OM and toward the pelagic end of the isotopic gradient.

Stable isotope mixing models based on δ^13^C and δ^15^N values indicated that benthic-derived sources generally dominated the diets of resident consumers (Figure 4). *L. santolla*, *E. maclovinus*, and *Patagonotothen* sp. exhibited the highest reliance on macroalgal-derived production, with macroalgae accounting for more than 80% of their assimilated biomass. Similarly, *G. gregaria* (benthic) and *E. megalocyathus* were primarily supported by benthic pathways, with macroalgal contributions of approximately 73% and 65%, respectively, accompanied by moderate contributions from sediment-derived organic matter. In contrast, *S. fuegensis* showed comparatively lower and more uncertain reliance on macroalgae (57.1%; 95% CrI: 13.5–71.8%), indicating greater dietary flexibility within this group. *N. magellanica* and *P. australis* exhibited more mixed source contributions, with macroalgal contributions ranging from approximately 38% to 54%, while POM and sediment OM together accounted for approximately 50–65% of assimilated sources, suggesting trophic coupling between benthic and pelagic pathways. The pelagic stage of *G. gregaria* departed most clearly from this pattern, deriving most of its assimilated biomass from phytoplankton-derived material (~52%) rather than macroalgae (~28%).

Because these values represent posterior medians, their associated 95% credible intervals (Table S2) provide important information on the precision of the estimates across taxa. The macroalgal contribution to *L. santolla* was relatively tightly constrained (95% CrI: 72.3–94.8%), consistent with a strong contribution from this source. By contrast, for *P. australis*, the 95% credible intervals for all four sources were broad and substantially overlapped (macroalgae: 6.2–78.9%; sediment: 2.4–66.9%; POM: 2.1–57.3%; phytoplankton: 2.3–38.4%), indicating substantial uncertainty in the relative contributions of individual dietary sources and that the model could not confidently discriminate among them.

## 4. Discussion

Overall, our results are more consistent with the channel-coupling hypothesis than with the compartmentalization hypothesis. Isotopic niche segregation among consumers decreased with trophic position, while niche overlap and isotopic redundancy increased toward intermediate trophic levels, the qualitative signature predicted under H1, in which mobile, higher-order consumers progressively integrate energy originating from otherwise distinct benthic and pelagic pathways. Resource partitioning, by contrast, was concentrated among lower-trophic consumers, where isotopically distinct primary producers supported clearly differentiated basal pathways. Macroalgal-derived organic matter was the dominant source of assimilated carbon across resident taxa, whereas sediment OM, itself a mixture of reworked macroalgal, phytoplanktonic, and particulate inputs rather than an independent basal source, linked consumers to multiple primary producers simultaneously. Taken together, these patterns indicate that the FC-CMPA food web combines strong differentiation at its base with progressive integration of energy channels toward its intermediate trophic levels, rather than uniform segregation (H2) or uniform integration across the entire hierarchy.

### 4.1. Food-Web Structure and Trophic Organization

Stable isotope analyses revealed a well-defined trophic hierarchy comprising approximately four trophic levels from primary producers to top predators. This food-chain length is consistent with previous isotopic studies conducted in the Strait of Magellan and the Beagle Channel [25,57], suggesting that subantarctic coastal ecosystems share a broadly similar trophic architecture despite substantial environmental variability. Consistent with the channel-coupling hypothesis, the strength of isotopic segregation among consumers varied systematically with trophic position rather than remaining uniform across the food web. Consumers occupying trophic level 2 exhibited the widest carbon and nitrogen ranges and the highest trophic dispersion, indicating broad exploitation of available resources and strong niche differentiation at the base of the food web. These results suggest that the availability of contrasting primary production channels promotes resource partitioning among lower-level consumers, allowing species to exploit distinct prey assemblages and basal resource pools. Similar patterns of trophic specialization driven by resource diversity have been reported in other coastal ecosystems, where spatial variation in resource availability increases ecological opportunities for niche differentiation [5,58].

In contrast, isotopic overlap increased markedly among consumers occupying trophic level 3, which showed the lowest mean nearest-neighbor distance of any trophic level multiple pairwise overlap probabilities exceeding 60%. This pattern matches the specific signature predicted under H1: as trophic position increases, mobile consumers draw on resources already partly reworked and redistributed by lower trophic levels, so that isotopically distinct basal pathways converge into more similar assimilated signatures. Food-web theory predicts that consumers occupying intermediate trophic positions integrate energy originating from multiple prey assemblages and resource pools, generating greater isotopic similarity despite underlying dietary variation [59]. Notably, this convergence did not extend to the apex of the food web: overlap between the two trophic level 4 consumers remained low, indicating that redundancy is a property of intermediate, generalist trophic positions rather than a uniform trend across the whole hierarchy. Trophic diversity and trophic redundancy therefore appear to arise through different, trophic-position-specific mechanisms: resource partitioning promotes differentiation among lower-trophic consumers, trophic integration promotes convergence among intermediate consumers, and specialization re-emerges among top predators.

Species such as *G. gregaria* and *S. fuegensis* may contribute to this intermediate-level integration by transferring energy from multiple primary producers toward higher trophic consumers. This role matches food-web models developed specifically for this protected area, where *G. gregaria* and *S. fuegensis* were identified as key structural links connecting benthic and pelagic pathways within the FC-CMPA pelagic food web [24,25]. As resources become progressively integrated across trophic levels, isotopic convergence can also emerge among consumers occupying different ecological roles. This was evident in the substantial isotopic niche overlap observed between humpback whales and introduced salmonids. Although isotopic overlap alone cannot be interpreted as evidence of direct competition, it suggests partial reliance on shared forage resources and highlights the capacity of non-native salmonids to become incorporated into existing trophic pathways within the FC-CMPA ecosystem [60,61]. Seasonal and ontogenetic diet data for humpback whales in the Magellan Strait [62] points to substantial reliance on pelagic prey subject to interannual variability, which would be expected to further modulate the extent of this overlap. Importantly, the assimilation patterns inferred from mixing models describe the resident component of the food web and should not be extrapolated directly to highly mobile consumers. Species such as the humpback whale, the southern sea lion, the Magellanic penguin, the southern hake, and introduced salmonids forage over much larger spatial ranges than the resident taxa used in the mixing models, so their isotopic composition and trophic position more likely reflect broader movement and foraging patterns than direct coupling to the local benthic or pelagic pathways characterized here. This distinction is not in tension with the channel-partitioning pattern described for resident consumers: environmental heterogeneity generates isotopically distinct basal resource pools that structure resident consumers through direct source coupling, while it shapes highly mobile, higher-order consumers principally through their trophic position and foraging range rather than through coupling to a single local pathway. Collectively, these findings indicate that environmental heterogeneity within the FC-CMPA generates substantial diversity in basal resource pools among residents, but this diversity is progressively integrated, rather than propagated through parallel, fully segregated pathways, as it moves up the resident food web. Isotopic evidence indicates that resident consumers were supported predominantly by macroalgal-derived carbon, with detrital and pelagic organic matter, including sediment OM, which integrates reworked material from multiple primary producers rather than functioning as an independent source, providing secondary but non-negligible contributions. As organic matter is progressively reworked and transferred through successive trophic levels, these sources become increasingly integrated within consumer biomass, producing a food web characterized by simultaneous trophic differentiation at its base, detrital mediation through sediment, and cross-habitat connectivity mediated by species such as *G. gregaria*.

### 4.2. Basal Resource Assimilation and Trophic Integration

The central objective of this study was to assess the relative assimilation of benthic and pelagic basal resources by resident consumers within the FC-CMPA. Most resident consumers were predominantly supported by macroalgal-derived organic matter, highlighting benthic primary production as the dominant basal carbon pathway sustaining trophic transfer across the food web.

The isotopic separation between macroalgae and pelagic-derived sources was consistent with patterns reported across Patagonian fjords [63,64], and other temperate coastal ecosystems [65,66]. Macroalgae displayed relatively enriched δ^13^C signatures compared with phytoplankton and particulate organic matter, reflecting reduced isotopic discrimination during dissolved inorganic carbon uptake and species-specific differences in carbon acquisition pathways [66,67]. This isotopic separation generated a well-defined baseline framework that enabled robust discrimination among alternative sources supporting consumer production.

Despite the availability of isotopically distinct basal resources, macroalgal-derived production accounted for more than 50% of assimilated biomass across all resident trophic levels and exceeded 80% in several benthic and demersal taxa. These results reinforce a growing body of evidence demonstrating that macroalgal habitats function not only as structural ecosystem engineers but also as major contributors of organic carbon to coastal food webs through both direct consumption and detrital pathways [7,8,68,69]. Consequently, macroalgal-derived organic matter appears to represent the principal source of assimilated carbon supporting resident consumers within the FC-CMPA, rather than equivalent contributions from benthic and pelagic production.

Ontogenetic variation in *G. gregaria* provides the clearest direct test of channel coupling available in this dataset and represents an important exception to the predominantly benthic resource use observed among the other resident consumers. The benthic phase derived the majority of its assimilated resources from macroalgae, matching the general benthic pattern described above. The pelagic phase, by contrast, relied predominantly on phytoplankton-derived material—the reverse of the benthic phase’s dominant source. This shift is consistent with the general life history of galatheid crustaceans, whose early pelagic (planktonic) phase precedes benthic settlement [70], so that the isotopic reversal documented here likely tracks a genuine habitat transition rather than dietary flexibility within a single habitat. Because the two phases occupy contrasting compartments of the ecosystem with access to different resource pathways, and because both were sampled within the same population and study period, *G. gregaria* offers direct, individual-level evidence that a single species can physically move between, and sequentially assimilate, energy channels that otherwise remain isotopically segregated at the base of the food web. Consequently, the ontogenetic habitat shift in *G. gregaria* is not merely a localized exception but a concrete demonstration, at the individual level, of the channel-coupling process inferred indirectly from community-wide isotopic redundancy elsewhere in this study. This ecological flexibility is consistent with previous studies identifying galatheid crustaceans as important components and potential mediators of carbon transfer within subantarctic coastal ecosystems [71], and matches the structural role assigned to *G. gregaria* in food-web models developed specifically for the FC-CMPA [25].

### 4.3. Methodological Limitations and Scope of Inference

As with all bulk stable isotope approaches, uncertainty in trophic discrimination factors and isotopic baselines can affect estimates of trophic position and source contributions, particularly when baseline variability or species-specific discrimination factors are not fully constrained [13,14,72]. This uncertainty is especially relevant for mobile consumers, whose tissues may integrate resources acquired beyond the spatial extent of local reference organisms. Accordingly, trophic positions should be interpreted as indicators of relative food-web structure rather than absolute feeding levels. This limitation is further compounded by the evaluation of trophic structure and primary food sources at different ecological scales, requiring cautious interpretation when linking trophic-position estimates to resource-use patterns. Isotopic niche analyses incorporated both resident and mobile consumers, providing a regional perspective on trophic organization, niche differentiation, and connectivity across the broader assemblage. In contrast, resource assimilation was assessed primarily using resident consumers, whose isotopic composition more accurately reflects locally integrated basal resources and patterns of resource use within the FC-CMPA. Consequently, patterns of trophic overlap and niche structure should be interpreted as properties of the regional food web, whereas inferences regarding basal resource use apply specifically to the resident component of the ecosystem.

### 4.4. Future Perspectives

The patterns reported here for the FC-CMPA resident food web, pronounced resource partitioning at the base of the web and progressive integration toward intermediate trophic levels, were adequately characterized by the available sampling design and isotopic mixing models, but leave several avenues open for refinement. Future integration of seasonal sampling, compound-specific isotope analyses, and ecosystem modelling will help resolve whether the channel-coupling pattern described here (H1) is stable across seasons or shifts with the pronounced seasonality of pelagic production in Patagonian fjords and will further refine estimates of trophic connectivity and energy transfer across Patagonian coastal ecosystems.

From a conservation and management perspective, these findings carry direct implications for the FC-CMPA. Because macroalgal-derived organic matter underpins assimilated carbon across nearly all resident trophic levels, sustaining macroalgal forest cover should be treated as a priority for maintaining food-web stability within the protected area. Periodic isotopic monitoring of pelagic subsidies and sediment OM could serve as an early indicator of shifts in the relative importance of benthic and pelagic pathways, particularly given the sensitivity of pelagic production to interannual oceanographic variability [26,27]. The isotopic niche overlap detected between humpback whales and introduced salmonids further underscores the value of continued monitoring of non-native salmonid populations, given their demonstrated capacity to become incorporated into existing trophic pathways within the FC-CMPA [60,61]. Embedding these trophic considerations into the management of the protected area, for example through zoning or monitoring frameworks that account for both benthic and pelagic energy pathways, would help ensure that conservation measures reflect the way energy actually moves through this food web, rather than treating benthic and pelagic habitats as independent management units.

## 5. Conclusions

Stable isotope analyses reveal a trophic architecture within the FC-CMPA in which basal resource heterogeneity drives resource partitioning among lower-trophic consumers, while this differentiation gives way to isotopic redundancy and resource integration at intermediate trophic levels—consistent with the channel-coupling hypothesis (H1) rather than persistent compartmentalization (H2). Macroalgal production emerges as the dominant source of assimilated carbon across resident consumers, while sediment functions as an integrative pool linking multiple basal sources rather than as an independent pathway. Ontogenetic habitat shifts in G. gregaria provide direct, individual-level evidence that consumers can bridge otherwise isotopically distinct benthic and pelagic channels over their life cycle. Rather than operating as fully independent benthic and pelagic pathways, the FC-CMPA food web functions as a partially coupled trophic network, in which environmental heterogeneity generates diversity at its base without fragmenting the wider food web.

## Figures and Tables

**Figure 1:**
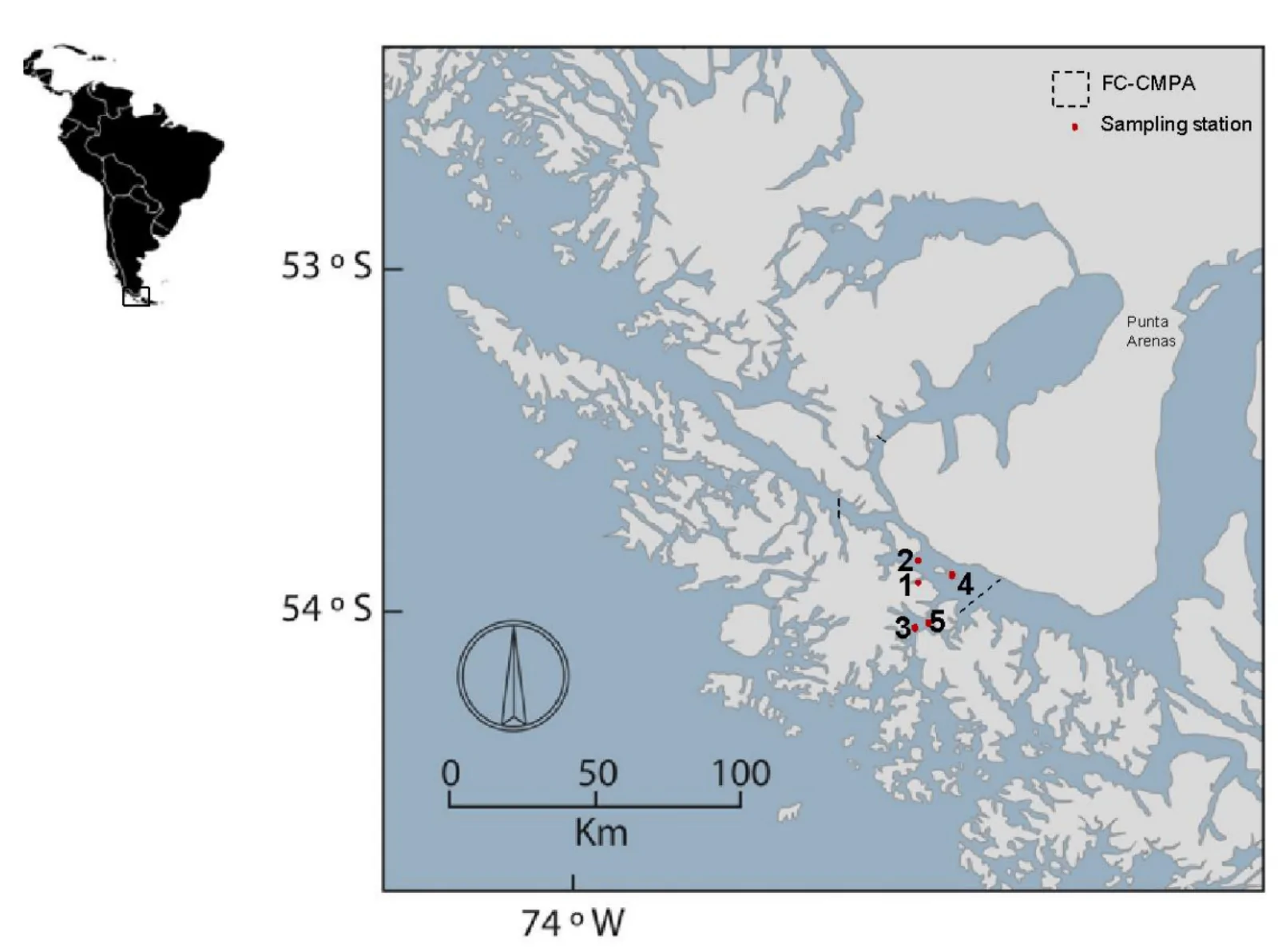
Site locations where consumers and primary food sources were sampled for stable isotopes analyses. Red circles indicate the five sites of study in the Francisco Coloane Coastal Marine Protected Area. 1. Choiseul Bay = *M. australis*; *E. maclovinus*, *Patagonotothen* sp., *P. australis*, *Oncorhynchus* sp.; 2. Southern Carlos III Island = *M. novaeangliae*; *G. gregaria* (benthic and pelagic); *L. santolla*; *A. utricularis*; *M. pyrifera*; 3. Paso Shag = *N. magellanica*, *P. purpuratus*, *S. fuegensis*, *E. megalocyathus*, *U. lactuca*, Phytoplankton, Superficial Sediment OM, POM. 4. Monmouth Island = *S. magellanicus*. 5. Cayetano Island = *O. byronia*.

**Figure 2:**
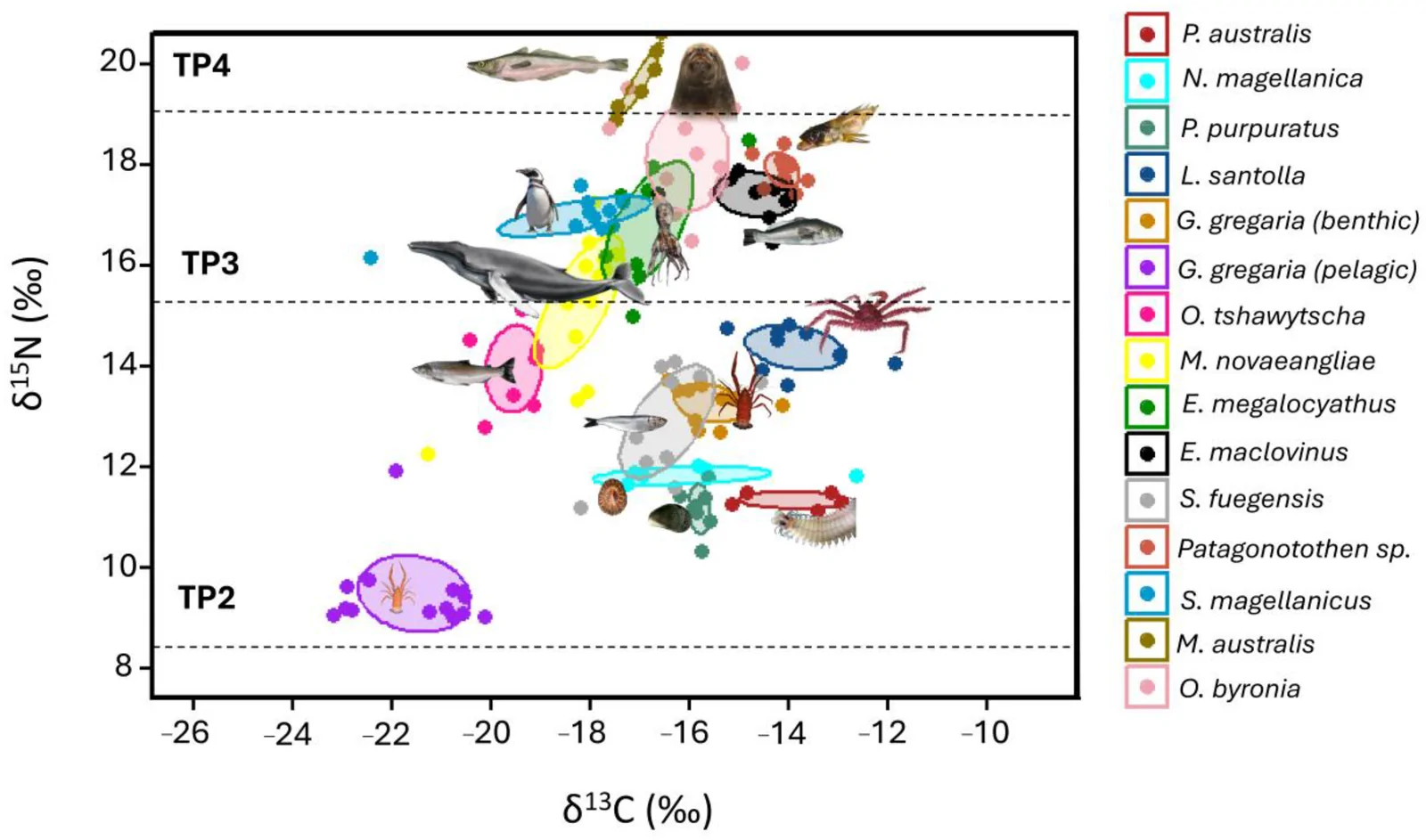
Trophic position (TP) and isotopic niche width of the consumer species sampled in the Francisco Coloane Coastal Marine Protected Area. The bivariate plot shows individual δ^13^C and δ^15^N values for each species (colored circles; see legend), with colored ellipses representing the standard ellipse area corrected for small sample size (SEAc). Horizontal dashed lines delimit trophic position bands (TP2–TP4) based on estimated trophic position.

**Figure 3:**
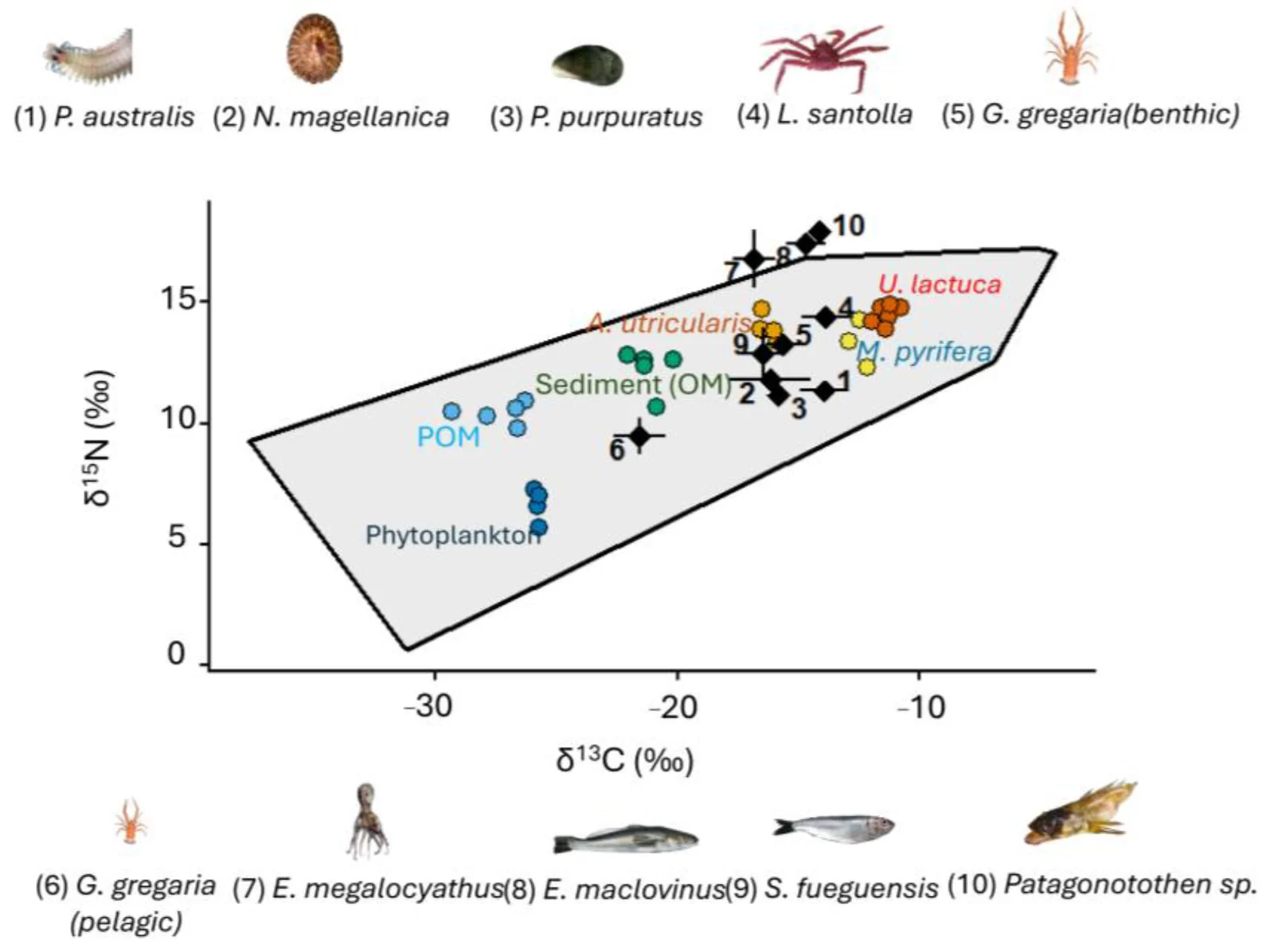
δ^13^C–δ^15^N isotopic biplot showing the distribution of resident consumers and primary food sources in isotopic space. Numbered black diamonds represent the mean isotopic values (±SD) of resident consumer taxa, while colored circles represent individual samples of primary food sources. Consumer isotopic values were corrected for trophic discrimination factors prior to analysis. The grey polygon represents the convex hull enclosing the source values and defines the feasible isotopic mixing space used in the SIMMR mixing model.

**Figure 4:**
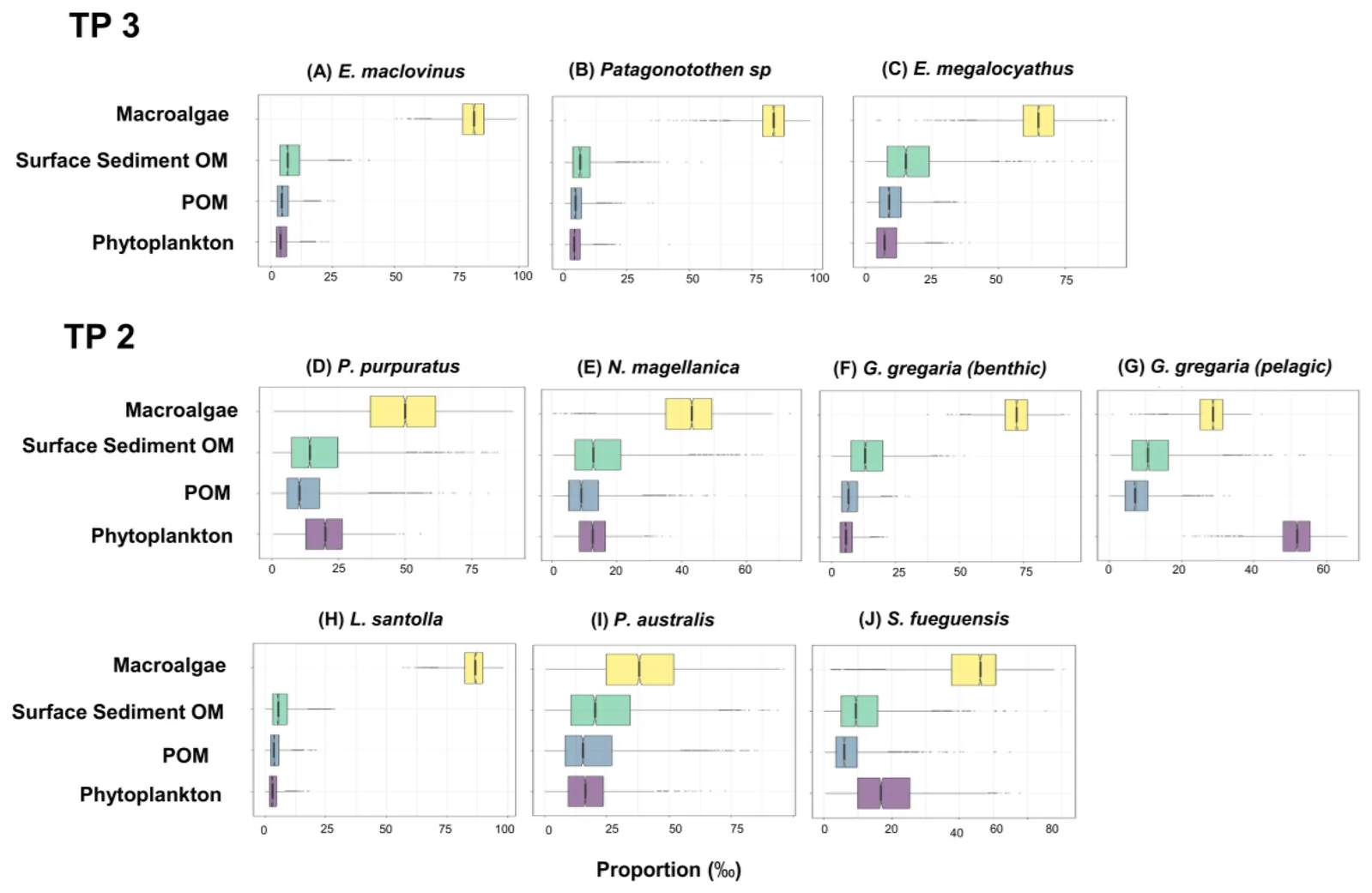
Boxplots representing food source assimilation (%) for each trophic level according to the SIAR mixing model (simmr). The centerline in the box is the median of all solutions, and the box is drawn around the 25th and 75th quartiles, thereby representing 50% of the solutions.

**Table 2:** Probability of niche overlap metric (%) for a specified region of 95%. Overlap probability is shown of the species displayed in rows onto those displayed in columns for the different trophic levels (TP). Probabilities > 60% are highlighted in bold.

**TP 4**	***M. australis***			***O. byronia***			
***M. australis***	–			46.4			
***O. byronia***	2.7			–			
**TP 3**	***E. maclovinus***	***Patagonotothen* sp.**	***E. megalocyathus***	***S. magellanicus***		***M. novaeangliae***	***Oncorhynchus* sp.**
***E. maclovinus***	–	27.9	28.1	22.2		3.9	0.0
***Patagonotothen* sp.**	**78.8**	–	10.4	10.8		0.0	0.0
***E. megalocyathus***	15.0	0.7	–	32.4		**63.7**	6.0
***S. magellanicus***	9.8	1.0	44.0	–		45.1	0.1
***M. novaeangliae***	1.4	0.0	60.0	13.7		–	32.2
***Oncorhynchus* sp.**	0.0	0.0	42.2	0.1		**98.4**	–
**TP 2**	***G. gregaria* (*benthic*)**	***N. magellanica***	***P. purpuratus***	***S. fuegensis***	***P. australis***	***L. santolla***	***G. gregaria* (*pelagic*)**
***G. gregaria* (*benthic*)**	–	0.9	0.9	**86.4**	0.1	6.2	0.0
***N. magellanica***	2.0	–	14.9	52.9	8.1	0.0	0.3
***P. purpuratus***	1.4	23.9	–	28.6	17.4	0.0	0.0
***S. fuegensis***	36.2	14.4	2.5	–	1.0	15.9	0.3
***P. australis***	0.3	12.3	5.5	4.5	–	0.0	0.0
***L. santolla***	3.9	0.0	0.0	42.8	0.0	–	0.0
***G. gregaria* (*pelagic*) **	0.0	0.1	0.0	0.3	0.0	0.0	–

**Table 3:** Values of Layman’s metrics by trophic levels. NR: δ^15^N range; CR: δ^13^C range; CD: mean distance to centroid; MNND: mean nearest-neighbor distance; SDNND: standard deviation of nearest-neighbor distance.

Trophic Level	NR	CR	CD	MNND	SDNND
**4**	1.5	1.0	0.9	1.8	0.0
**3**	2.6	4.8	1.8	1.0	0.5
**2**	3.2	5.7	2.0	1.8	1.2

## Data Availability

The original contributions presented in this study are included in the article; further inquiries can be directed to the corresponding authors.

## Supplementary Materials

The following supporting information can be downloaded at https://www.mdpi.com/article/10.3390/biology15191699/s1: Table S1: δ^13^C and δ^15^N values of basal food sources and pairwise comparisons of isotopic heterogeneity; Table S2: Posterior mean, SD, median, and 95% credible interval of primary food-source contributions to consumer diets from the Bayesian mixing model.
