# Supplementary material for: From Basal Sources to Top Predators: Isotopic Evidence of Trophic Organization in the Francisco Coloane Coastal Marine Protected Area
Source: Biology (Basel). 2026 Sep 23;15(19):1699. doi: 10.3390/biology15191699 (PMC13663473; doi:10.3390/biology15191699)
Supplement: Supplementary file 1 [file biology-15-01699-s001.zip › biology-4523020-supplementary.pdf]

Table S1. $\delta^{13}C$ and $\delta^{15}N$ values (mean ± SD) of the basal food sources. Superscript letters denote the results of Kruskal–Wallis and Dunn's post hoc pairwise comparisons (Benjamini–Hochberg correction, $\alpha = 0.05$) used to assess isotopic heterogeneity among sources; within each isotope, sources sharing no letter differ significantly ($p < 0.05$).

| **Source** | **n** | **δ13C (‰) ± SD** | **δ15N (‰) ± SD** |
|---|---|---|---|
| ***Ulva lactuca*** | 6 | -11.87 ± 0.37$^{a}$ | 11.09 ± 0.38$^{a}$ |
| ***Macrocystis pyrifera*** | 5 | -12.70 ± 0.53$^{ab}$ | 10.34 ± 0.90$^{ab}$ |
| ***Adenocystis utricularis*** | 5 | -16.82 ± 0.32$^{abc}$ | 10.50 ± 0.48$^{ab}$ |
| **Sediment organic matter** | 5 | -21.71 ± 0.69$^{bcd}$ | 8.84 ± 0.86$^{bc}$ |
| **Phytoplankton** | 5 | -26.23 ± 0.09$^{cd}$ | 3.31 ± 0.60$^{c}$ |
| **POM** | 5 | -27.86 ± 1.25$^{d}$ | 7.00 ± 0.41$^{bc}$ |

Table S2. Posterior estimates of primary food-source contributions to consumer diets. Posterior mean, standard deviation (SD), median, and 95% credible interval (CrI) are reported for each food source and consumer based on the Bayesian mixing model

| Consumer | Primary food source | Mean (%) | SD (%) | Median (%) | 95% CrI |
|---|---|---|---|---|---|
| ***E. maclovinus*** | Phytoplankton | 4.78 | 3.24 | 4.07 | 0.68–12.88 |
| | MOP | 5.33 | 3.42 | 4.66 | 0.84–13.58 |
| | Sediment | 8.35 | 5.94 | 6.89 | 0.97–23.30 |
| | Macroalgae | 81.53 | 6.49 | 82.01 | 67.31–92.76 |
| ***P. australis*** | Phytoplankton | 17.23 | 9.84 | 16.30 | 2.32–38.37 |
| | MOP | 19.45 | 14.80 | 15.37 | 2.14–57.26 |
| | Sediment | 24.36 | 17.56 | 20.18 | 2.38–66.88 |
| | Macroalgae | 38.96 | 19.13 | 38.06 | 6.19–78.92 |
| ***P. purpuratus*** | Phytoplankton | 19.89 | 9.05 | 20.10 | 3.70–36.80 |
| | MOP | 13.51 | 10.95 | 10.38 | 1.54–43.21 |
| | Sediment | 18.09 | 13.82 | 14.27 | 2.07–53.61 |
| | Macroalgae | 48.51 | 16.77 | 50.01 | 12.15–76.24 |
| ***N. magellanica*** | Phytoplankton | 15.62 | 6.96 | 15.48 | 3.34–29.33 |
| | MOP | 13.26 | 9.28 | 11.07 | 1.84–36.14 |
| | Sediment | 19.15 | 13.82 | 15.91 | 2.10–53.59 |
| | Macroalgae | 51.97 | 13.60 | 53.81 | 20.68–74.01 |
| ***G. gregaria (benthic)*** | Phytoplankton | 6.15 | 3.44 | 5.62 | 1.21–14.25 |
| | MOP | 7.43 | 4.43 | 6.58 | 1.29–17.67 |
| | Sediment | 14.06 | 8.24 | 12.78 | 1.95–31.97 |
| | Macroalgae | 72.36 | 6.46 | 72.51 | 58.67–84.27 |
| ***G. gregaria (pelagic)*** | Phytoplankton | 51.45 | 5.69 | 51.78 | 39.06–61.13 |

| | | | | | |
|---|---|---|---|---|---|
| | MOP | 8.17 | 4.84 | 7.25 | 1.50–19.53 |
| | Sediment | 12.33 | 7.82 | 10.86 | 1.92–31.01 |
| | Macroalgae | 28.05 | 4.68 | 28.62 | 17.43–35.67 |
| ***Patagonotothen sp.*** | Phytoplankton | 4.60 | 3.21 | 3.89 | 0.68–12.69 |
| | MOP | 4.99 | 3.46 | 4.28 | 0.72–13.40 |
| | Sediment | 7.46 | 5.77 | 6.13 | 0.82–21.56 |
| | Macroalgae | 82.96 | 7.25 | 83.61 | 67.26–94.29 |
| ***E. megalocyathus*** | Phytoplankton | 8.45 | 5.60 | 7.23 | 1.24–22.34 |
| | MOP | 9.85 | 6.05 | 8.82 | 1.48–23.86 |
| | Sediment | 17.21 | 11.40 | 15.18 | 2.01–44.08 |
| | Macroalgae | 64.48 | 9.56 | 64.96 | 43.23–81.45 |
| ***S. fuegensis*** | Phytoplankton | 23.61 | 14.23 | 21.00 | 3.58–54.72 |
| | MOP | 9.31 | 7.15 | 7.54 | 1.33–26.64 |
| | Sediment | 14.47 | 10.52 | 11.95 | 1.77–39.84 |
| | Macroalgae | 52.61 | 15.34 | 57.14 | 13.48–71.83 |
| ***L. santolla*** | Phytoplankton | 3.42 | 2.31 | 2.89 | 0.58–9.41 |
| | MOP | 4.21 | 2.82 | 3.57 | 0.65–11.05 |
| | Sediment | 6.56 | 4.90 | 5.25 | 0.71–18.90 |
| | Macroalgae | 85.81 | 5.79 | 86.65 | 72.28–94.84 |
